# Chemoradiotherapy for limited-stage small-cell lung cancer and interstitial lung abnormalities

**DOI:** 10.1186/s13014-021-01780-y

**Published:** 2021-03-17

**Authors:** Haruki Kobayashi, Kazushige Wakuda, Tateaki Naito, Nobuaki Mamesaya, Shota Omori, Akira Ono, Hirotsugu Kenmotsu, Haruyasu Murakami, Masahiro Endo, Hideyuki Harada, Yasuhiro Gon, Toshiaki Takahashi

**Affiliations:** 1 Division of Thoracic Oncology, Shizuoka Cancer Center, 1007 Shimonagakubo, Nagaizumi-cho, Sunto-gun, Shizuoka 411-8777 Japan; 2 Division of Diagnostic Radiology, Shizuoka Cancer Center, Shizuoka, Japan; 3 Division of Radiation and Proton Therapy, Shizuoka Cancer Center, Shizuoka, Japan; 4 Division of Respiratory Medicine, Nihon University School of Medicine, Tokyo, Japan

**Keywords:** Limited-stage small-cell lung cancer, Chemoradiotherapy, Interstitial lung disease, Interstitial lung abnormalities, Radiation pneumonitis

## Abstract

**Background:**

Patients with lung cancer and interstitial lung disease treated with radiotherapy are at risk of developing radiation pneumonitis. However, the association between interstitial lung abnormalities (ILAs) and radiation pneumonitis in patients with limited-stage small-cell lung cancer (LS-SCLC) remains unclear. Furthermore, the prognosis is uncertain for patients with SCLC and ILAs treated with chemoradiotherapy. We investigated the impact of ILAs on radiation pneumonitis and assessed the prognosis of patients with LS-SCLC and ILAs treated with chemoradiotherapy.

**Methods:**

We retrospectively reviewed the medical records of 149 patients with LS-SCLC who received first-line treatment between January 2009 and December 2016.

**Results:**

In the univariate analysis, the patients with ILAs showed a higher incidence rate of radiation pneumonitis compared with those without ILAs (64% vs. 10%, *P* < 0.001). Multivariate analysis confirmed that ILAs were significantly associated with the incidence of radiation pneumonitis. In the univariate analysis, patients with ILAs showed poorer overall survival than those without ILAs (median, 18.9 vs. 67.9 months, *P* = 0.0338). Multivariate analysis showed that ILAs were a significant independent negative prognostic factor. However, the 2-year and 5-year survival rates for the patients with ILAs treated with chemoradiotherapy were 36% and 26%, respectively, and 8% and 0%, respectively, for those treated with chemotherapy alone.

**Conclusions:**

ILAs were found to be a predictive factor for radiation pneumonitis in patients with LS-SCLC treated with chemoradiotherapy. Patients with LS-SCLC and ILAs who were treated with chemoradiotherapy had both the possibility of long-term survival and risk of radiation pneumonitis.

**Supplementary Information:**

The online version contains supplementary material available at 10.1186/s13014-021-01780-y.

## Background

The standard treatment for patients with limited-stage small-cell lung cancer (LS-SCLC) is concurrent chemoradiotherapy (CCRT) [1–3]. However, patients with lung cancer and interstitial lung disease (ILD) treated with radiotherapy have been reported to be at risk of developing radiation pneumonitis (RP) [4]. Therefore, we usually avoid prescribing radiotherapy to such patients.

Due to the recent expansion of chest computed tomography (CT) scans, slight concomitant interstitial lung changes have been detected in patients with emphysema and in lung cancer-screening populations [5, 6]. These slight changes have been classified as interstitial lung abnormalities (ILAs), defined as non-dependent abnormalities affecting more than 5% of any lung zone [7, 8].

Our previous study showed that ILAs were a risk factor for RP in patients with non-small-cell lung cancer (NSCLC) treated with CCRT [9]. However, the association between pre-existing radiological ILAs and RP in patients with SCLC remains unclear. Additionally, the prognosis for patients with SCLC and ILAs treated with CCRT is uncertain because the slight radiological findings of ILAs are often missed. Therefore, this study investigated the association between ILAs and RP in patients with LS-SCLC and ILAs treated with CCRT at our institution and evaluated their prognosis.

## Methods

### Patients and exclusion criteria

The medical records of patients with LS-SCLC who underwent chemotherapy, radiotherapy, or surgery as first-line treatment at our institution in Japan between January 2009 and December 2016 were retrospectively reviewed. The tumor, nodes, metastasis (TNM) stage was evaluated based on the 7th edition of the TNM classification of lung cancer [10]. Early CCRT as the first-line treatment was defined as the administration of radiotherapy within 14 days after chemotherapy treatment.

The initial analysis included the patients with LS-SCLC who were treated with early CCRT as the first-line treatment. We then compared the patients with LS-SCLC and ILAs treated with concurrent or sequential chemoradiotherapy (CRT) and chemotherapy alone as the first-line treatment. The comparison excluded patients with contralateral hilar lymph node metastasis treated with chemotherapy alone as the first-line treatment.

Patients who had previously received any chemotherapy or chest radiotherapy were excluded from this study as well as those who were not followed up within 30 days after the final day of the first-line treatment.

The study protocol was performed in accordance with the Declaration of Helsinki.

### Treatment and patient evaluation

Early or late concurrent radiotherapy treatment was performed for the patients with LS-SCLC included in this study with the first cycle of chemotherapy or sequentially after four cycles of chemotherapy. In most cases, the total planned dose was 45 Gy in twice-daily fractions or 50 Gy in a once-daily fraction [11]. The initial field for the patients who received the radiotherapy sequentially was based on the post-chemotherapy treatment tumor volume. The timing and prescribed dose of radiotherapy was determined by the physician in charge. All patients were required to undergo a chest CT to facilitate treatment planning. The gross tumor volume (GTV) of the primary tumor (primary GTV) was delineated in the pulmonary windows, and the nodal involvement (nodal GTV) was delineated in the mediastinal windows. The clinical target volume (CTV) initially included the primary and nodal GTVs, the ipsilateral hilum, and the elective mediastinum, for which the lower border was 3.0 cm below the carina. The dose was up to 40 Gy in a once-daily fraction of 2 Gy or 30 Gy in twice-daily fractions of 1.5 Gy per fraction. Thereafter, the CTV only included the primary GTV and nodal GTV. The planning target volume was the CTV plus a margin that ensured that the planned dose was actually delivered to the CTV. After the radiotherapy, prophylactic cranial irradiation was administered to patients with a complete or near-complete response, represented by a scar-like shadow on chest CT, if the physician in charge judged that this would be beneficial to the patient. The prophylactic cranial irradiation consisted of 25 Gy in 10 fractions [12].

ILAs were defined as non-dependent abnormalities affecting more than 5% of any lung zone [7], excluding cases of infectious lung disease or drug-induced pneumonia. The existence of ILAs in the chest CT scans acquired before the treatment was evaluated by one radiologist (a member of the ILD Committee of Drug-induced Pneumonitis) and two pulmonologists (in their 12th and 26th years of practice as pulmonologists, respectively) blinded to the patient outcomes. The area of ILAs as a proportion of the area of a lung zone was measured every 5% by visual evaluation.

The development of RP or the acute exacerbation of ILAs within 1 year after the last day of irradiation was considered an event of radiation-related pneumonitis (RRP). The definition of RRP included any acute respiratory event characterized by new bilateral ground-glass opacification or consolidation that was not entirely explained by an infectious disease, cardiac failure, or fluid overload [13]. An RRP event was considered as an RRP of grade 2 or higher with steroid administration because of dyspnea and decreased transcutaneous oxygen saturation, based on the National Cancer Institute Common Terminology Criteria version 4.0 [14]. If a patient treated with intravenous steroid pulse therapy died within 45 days after the last day of irradiation, the death was considered to have been caused by RRP.

### Statistical analysis

Categorical variables were analyzed using the Fisher exact test. The Cox proportional hazards approach was used in univariate and multivariate analyses of the incidence of RRP, progression-free survival (PFS), and overall survival (OS). OS was defined as the time from the start of the platinum-based chemotherapy as first-line treatment to death. PFS was calculated from the start of the platinum-based chemotherapy as first-line treatment to the date of disease progression or death from any cause. The end date for the survival analyses was defined as February 25, 2019. Additionally, 2- and 5-year survival rates were estimated using Kaplan–Meier survival probabilities, and the event times were estimated using the Kaplan–Meier method. The log-rank test was used to compare the cumulative survival between groups. All *P* values were two-sided, with values < 0.05 considered statistically significant. The statistical analyses were performed using JMP v11.2.0 software (SAS Institute, Cary, NC, USA).

## Results

### Patient characteristics

**Fig. 1:**
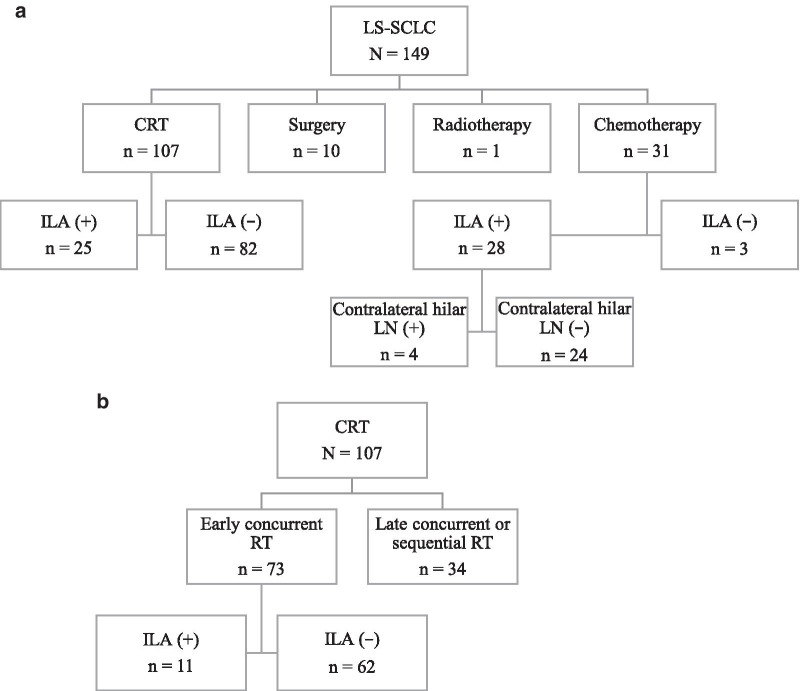
CONSORT diagrams of the study population. **a** CONSORT diagram of all the patients with LS-SCLC included in our study. **b** CONSORT diagram of the patients with LS-SCLC treated with CRT in our study. LS-SCLC, limited-stage small-cell lung cancer; CRT, chemoradiotherapy; ILA, interstitial lung abnormality; RT, radiotherapy; LN, lymph node

During the defined study period, a total of 149 patients diagnosed with LS-SCLC underwent first-line treatment, including CRT (n = 107), surgery (n = 10), chemotherapy alone (n = 31), and radiotherapy alone (n = 1) (Fig. 1a). Of them, 56 patients (38%) had concomitant ILAs. The 107 patients treated with CRT were divided into early CCRT (n = 73) and late CCRT or sequential radiotherapy (n = 34) groups (Fig. 1b).

The baseline characteristics of the 73 patients treated with early CCRT are shown in Table 1. Of them, 11 (15%) had concomitant ILAs. The ILAs accounted for 5% of the area of a lung zone in seven (64%) cases and 10% of the area of a lung zone in the remaining four cases (36%) (typical CT images of ILAs are shown in Additional file 1: Fig. S1). There were no significant differences in the characteristics of LS-SCLC patients between the ILA and non-ILA groups (Additional file 2: Table S1).

**Table 1:** Baseline characteristics of 73 patients with LS-SCLC treated with early CCRT

Variable	Result
Age, median (range), years ≥ 70/ < 70 years	66 (34–77) 19/54
Sex, male/female	55/18
ECOG-PS, 0/1/2	42/28/3
Clinical stage, I/II/III^a^	0/15/58
Brinkmann index, ≥ 400/ < 400	71/2
Preexistence of ILAs, yes/no	11/62
V20, median (range), %	24 (13–36) (n = 72)
Number of patients receiving AHF	72
Number of patients receiving PCI	45
Chemotherapy regimen, CDDP/CBDCA	72/1

^a^Clinical staging according to the 7th edition of the TNM classification of lung cancer [9]. LS-SCLC, limited-stage small-cell lung cancer

CCRT, concurrent chemoradiotherapy; ECOG-PS, Eastern Cooperative Oncology Group performance status; ILA, interstitial lung abnormality; V20, percentage of normal lung receiving at least 20 Gy; AHF, accelerated hyperfractionation; PCI, prophylactic cranial irradiation; CDDP, cisplatin; CBDCA, carboplatin

The median follow-up duration for the censored cases was 60.4 months (range, 12.4–120.6 months). One patient with ILAs treated with early CCRT could not be administered the prescribed dose to cure because of RRP development.

### Prognosis

**Fig. 2:**
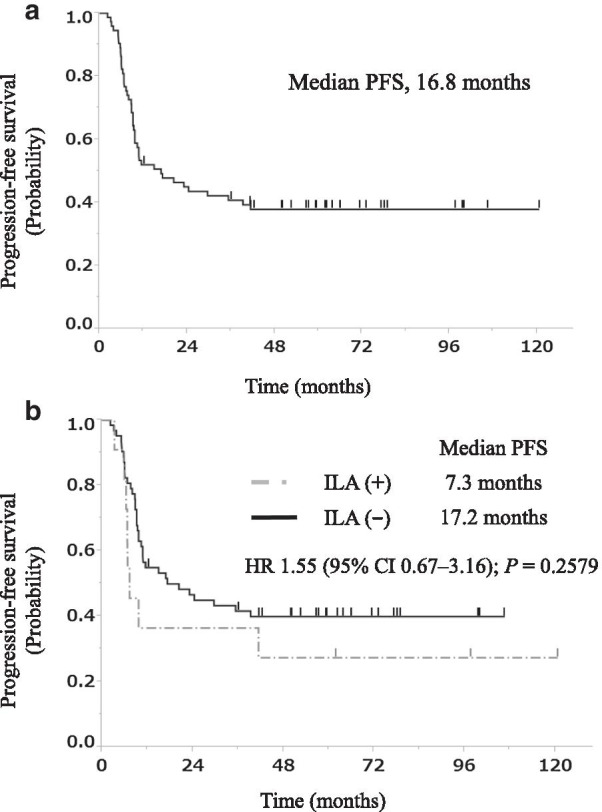
PFS curves of our study population. **a** PFS curve for the 73 patients with LS-SCLC treated with early CCRT. **b** PFS curves for these patients with ILAs (n = 11) and without ILAs (n = 62). PFS, progression-free survival; HR, hazard ratio; CI, confidence interval

The median PFS at the first-line treatment for all the patients with LS-SCLC treated with early CCRT was 16.8 months (Fig. 2a). There were no significant differences between the categorical variables shown in Table 2, in which the preexistence of ILAs was included (median PFS, 17.2 vs. 7.3 months, *P* = 0.2579; Fig. 2b).

**Table 2:** Univariate analysis of PFS in 73 patients with LS-SCLC treated with early CCRT

Variable	Univariate analysis		
	Relative risk	95% CI	*P *value
Sex, male	1.37	0.69–3.02	0.3983
Age, ≥ 70 years	0.80	0.39–1.53	0.5190
ECOG-PS = 0	0.81	0.45–1.48	0.4867
Clinical stage III	1.22	0.61–2.69	0.5967
Pre-existing ILAs	1.55	0.67–3.16	0.2579

PFS, progression-free survival; LS-SCLC, limited-stage small-cell lung cancer; CCRT, concurrent chemoradiotherapy; CI, confidence interval; ECOG-PS, Eastern Cooperative Oncology Group performance status; ILA, interstitial lung abnormality

**Fig. 3:**
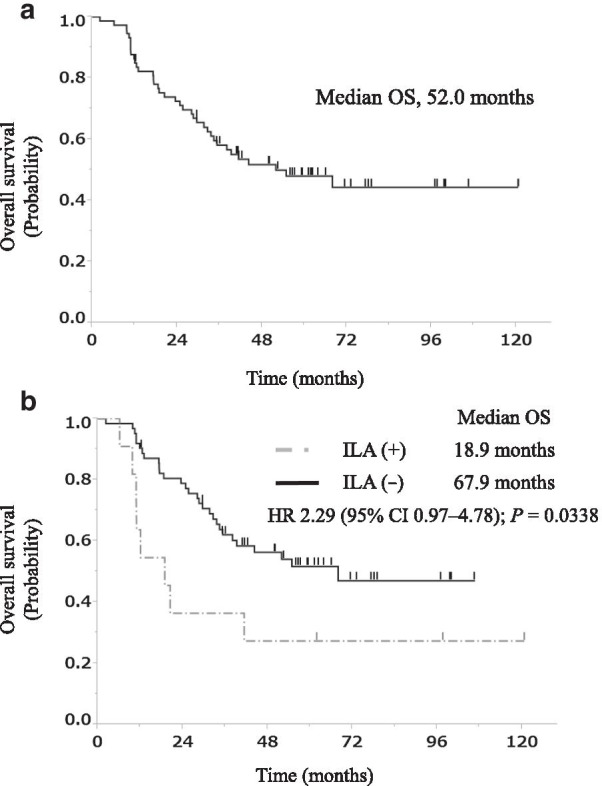
OS curves of our study population. **a** OS curve for 73 patients with LS-SCLC treated with early CCRT. **b** OS curves for these patients with ILAs (n = 11) and without ILAs (n = 62). CRT, chemoradiotherapy; ILA, interstitial lung abnormality; OS, overall survival; HR, hazard ratio; CI, confidence interval

For all the patients treated with early CCRT, the 2- and 5-year survival rates at the first-line treatment were 52% and 48%, respectively, and the median OS was 52.0 months (Fig. 3a). Table 3 summarizes the results of the univariate and multivariate analyses of OS for these patients. In the univariate analysis, OS was poorer for the patients with pre-existing ILAs than for those without ILAs (median OS, 18.9 vs. 67.9 months, *P* = 0.0338; Fig. 3b). The multivariate analysis showed that pre-existing ILAs were a significant independent negative prognostic factor, with a hazard ratio (HR) of 2.51 (95% confidence interval [CI], 1.02–5.62; *P* = 0.0460). The 2- and 5-year survival rates for the patients with ILAs were 35% and 27%, respectively, whereas those for the patients without ILAs were 78% and 52%, respectively. None of the patients died from RRP.

**Table 3:** Univariate and multivariate analyses of OS in 73 patients with LS-SCLC treated with early CCRT

Variable	Univariate analysis			Multivariate analysis		
	Relative risk	95% CI	*P *value	Relative risk	95% CI	*P *value
Sex, male	1.77	0.79–4.71	0.1933	–	–	
Age ≥ 70 years	2.28	1.18–4.20	0.8471	0.88	0.39–1.84	0.7511
ECOG-PS = 0	0.86	0.45–1.67	0.6398	0.83	0.42–1.64	0.5840
Clinical stage III	0.95	0.45–2.23	0.8988	0.74	0.32–1.85	0.5002
Pre-existing ILAs	2.29	0.97–4.78	0.0338	2.51	1.02–5.62	0.0460

OS, overall survival; LS-SCLC, limited-stage small-cell lung cancer; CCRT, concurrent chemoradiotherapy; CI, confidence interval; ECOG-PS, Eastern Cooperative Oncology Group performance status; ILA, interstitial lung abnormality

### Incidence of RRP

Of the 73 patients with LS-SCLC treated with early CCRT, 13 (18%; 95% CI, 11–28%) suffered RRP of grade 2 or worse (grade 2, n = 10; grade 3, n = 3) and required steroid treatment within 1 year after the final irradiation. None of the patients had RRP higher than grade 3. Table 4 summarizes the univariate and multivariate analyses for the incidence of RRP. In the univariate analyses, the patients with pre-existing ILAs showed a higher incidence rate of RRP compared with those without ILAs (64% vs. 10%, *P* < 0.001). The multivariate analysis showed that pre-existing ILAs were significantly associated with the incidence of RRP (HR, 27.7; 95% CI, 3.73–105; *P* < 0.001). Among the 11 patients with ILAs, 5 (45%) experienced grade 2 RRP, and 2 (18%) experienced grade 3 RRP. In contrast, of the 62 patients without ILAs, only 5 (8%) and 1 (2%) experienced RRP of grades 2 and 3, respectively.

**Table 4:** Univariate and multivariate analyses of RRP in 73 patients with LS-SCLC treated with early CCRT

Variable	Univariate analysis			Multivariate analysis		
	HR	95% CI	*P *value	HR	95% CI	*P *value
Sex male	4.74	0.83–89.7	0.0850	5.29	0.68–123	0.1221
Age ≥ 70 years	1.33	0.32–4.77	0.6717	–	–	–
ECOG-PS = 0	1.22	0.36–4.46	0.7464	–	–	–
V20 ≥ 25%	2.03	0.61–7.42	0.2526	3.64	0.82–20.9	0.0900
Pre-existing ILAs	16.3	3.88–80.3	0.0001	27.7	3.73–105	0.0002

RRP, radiation-related pneumonitis; LS-SCLC, limited-stage small-cell lung cancer; CCRT, concurrent chemoradiotherapy; HR, hazard ratio; CI, confidence interval; ECOG-PS, Eastern Cooperative Oncology Group performance status; V20, percentage of normal lung receiving at least 20 Gy; ILA, interstitial lung abnormality;

We analyzed the relationship between the incidence of RRP and the lung volume that was planned to receive at least 20 Gy (V20) of radiation therapy (Additional file 3: Fig. S2a). There was no difference in the lung V20 between the presence or absence of RRP (*P* = 0.2097). Additionally, according to the QUANTEC criteria, we analyzed the relationship of the existence of ILAs with lung V20 planned before radiation therapy (Additional file 3: Fig. S2b) and with mean lung dose (MLD) planned before radiation therapy (Additional file 3: Fig. S2c) due to radiation dose constraints. As shown in Additional file 3: Fig. S2b and S2c, RRP occurred even in patients with ILAs who received lower-dose radiation therapy with respect to the lung V20 or MLD.

In some of the patients who experienced RRP, the standard chemotherapy regimens recommended by the 2020 Japanese Lung Cancer Society Guidelines [15], such as amrubicin or weekly cisplatin plus etoposide plus irinotecan, were not chosen in an effort to avoid chemotherapy-induced pneumonitis.

### Impact of radiation therapy

We explored the impact of adding radiation therapy to chemotherapy for patients with LS-SCLC and ILAs. A total of 56 patients diagnosed with LS-SCLC and ILAs underwent first-line treatment of whom 25 received CRT, including early or late concurrent or sequential radiation therapy. All 24 patients without contralateral hilar lymph node (N3) involvement received chemotherapy alone (Fig. 1a). The characteristics of these patients are summarized in Table 5. There were no statistically significant differences in age, sex, Eastern Cooperative Oncology Group performance status, clinical stage, or smoking status between these two groups. However, compared with the patients treated with chemotherapy alone as the first-line treatment, the ILAs of those treated with CRT tended to cover a lower proportion of the area of any lung zone (*P* = 0.0106).

**Table 5:** Baseline characteristics of patients with LS-SCLC and ILA in this study

Variable	First-line treatment		
	Chemoradiotherapy	Chemotherapy	*P* value
	(n = 25)	(n = 24)	
Age, ≥ 70/ < 70 years	16/9	18/6	0.5380
Sex, male/female	21/4	20/4	1.0000
ECOG-PS, 0/1/2 (0 vs. 1 or 2)	15/10/0	10/11/3	0.2578
Clinical stage^a^, I/II/III (I vs. II/III)	1/1/23	1/5/18	1.0000
Brinkmann index, ≥ 400/ < 400	24/1	23/1	1.0000
Proportion of ILA area of any lung zone, 5%/10%/15–30%/30–50% (5%/10% vs. 15–30%/30–50%)	13/11/1/0	9/7/7/1	0.0106
V20, median (range), %	22 (10–32)		

^a^Clinical staging was according to the 7th edition of the TNM classification of lung cancer [9]. LS-SCLC, limited-stage small-cell lung cancer

ECOG-PS, Eastern Cooperative Oncology Group performance status; ILA, interstitial lung abnormality; V20, percentage of normal lung receiving at least 20 Gy

**Fig. 4:**
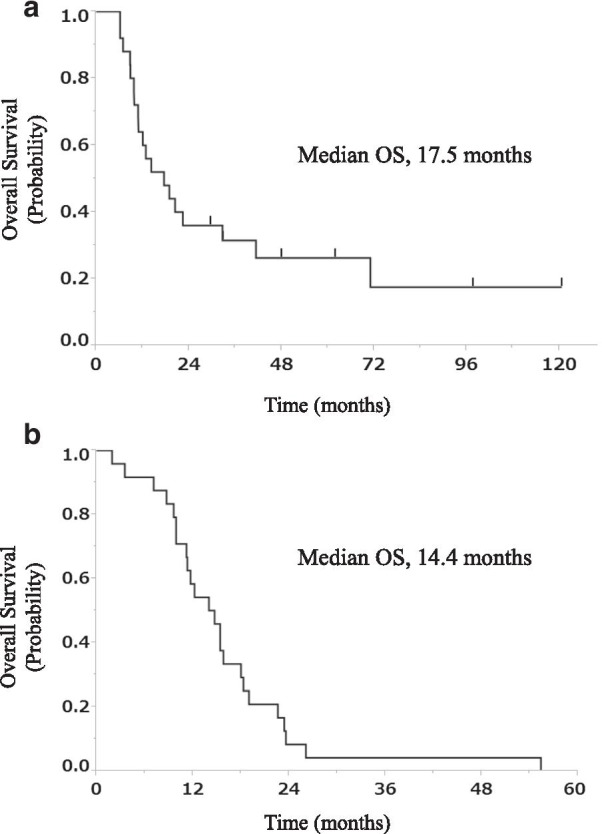
OS curve for patients with LS-SCLC and ILAs. **a** OS curve for 25 patients with LS-SCLC and ILAs treated with CRT. **b** OS curve for 24 patients with LS-SCLC and ILAs treated with chemotherapy alone. OS, overall survival

The median OS at the first-line treatment was 17.5 months for the patients treated with CRT and 14.4 months for those treated with chemotherapy alone (Fig. 4). The 2-year and 5-year survival rates for the patients treated with CRT were 36% and 26%, respectively, and 8% and 0%, respectively, for those treated with chemotherapy alone.

## Discussion

Our study results showed that pre-existing radiological ILAs were a risk factor for RRP in patients with LS-SCLC. Furthermore, the patients with LS-SCLC who had ILAs and were treated with early CCRT had a poorer prognosis than those without ILAs. However, some patients with LS-SCLC and ILAs treated with CRT achieved long-term survival.

We retrospectively reviewed the records of 73 patients with LS-SCLC treated with early CCRT, of which 11 (15%) had concomitant ILAs. Pre-existing ILAs were significantly associated with the incidence of RRP, regardless of lung V20 or MLD. This suggests that the diagnosis of ILAs could be a predictive factor for RRP in patients with LS-SCLC treated with CCRT, although severe RRP (grade 4 or more) was not observed. The frequency of RRP was similar to that in our previous study of patients with NSCLC and ILA treated with CRT [9]. ILAs may be of similar importance as ILD in predicting RRP because a portion of ILAs were reported to progress to ILD [16]. Although previous studies reported the unfavorable impact of ILAs on RP in patients with lung cancer receiving thoracic radiotherapy, their study cohorts were heterogeneous populations with regard to tumor stage, total radiation dose, chemotherapy regimen, and timing of radiotherapy [17, 18]. In contrast, in our study, most patients received a treatment of etoposide and cisplatin plus concurrent accelerated hyperfractionated thoracic radiotherapy, which is the standard treatment recommended by the 2020 Japanese Lung Cancer Society Guidelines [15]. Few studies have focused on the risk of RRP for patients with LS-SCLC treated with radiotherapy. Therefore, our study findings are crucial to understanding the risk of RRP before patients with LS-SCLC and ILA receive CRT.

We analyzed the prognosis of the patients with LS-SCLC treated with early CCRT. There was no difference in PFS between the patients with ILAs and those without ILAs. On the other hand, pre-existing ILAs were a significant independent negative prognostic factor for OS. There was no difference between the patients with and without ILAs in the rate of second-line chemotherapy given at the time of SCLC relapse. However, because of RRP, fewer patients with ILAs were able to receive the regimen recommended by the 2020 Japanese Lung Cancer Society Guidelines. It has also been reported that patients with ILAs without cancer have a poor prognosis [8, 16]. Therefore, other than SCLC, increased all-cause mortality can be due to ILAs in this population.

This study included a total of 149 patients diagnosed with LS-SCLC who underwent first-line treatment, of whom 56 (38%) had concomitant ILAs. Concomitant ILAs have been observed in approximately 10–20% of the patients included in trials concerning chronic obstructive pulmonary disease (the COPD Gene study [5, 19], ECLIPSE Study [20], and AGES-Reykjavik Study [21]), a study of screening for lung cancer using low-dose helical CT scans (the National Lung Screening Trial [6, 22]), and a study to identify common factors or characteristics that contribute to cardiovascular disease (Framingham Heart Study) [8, 16, 23]. The proportion of patients with concomitant ILAs in our study was substantially greater than in those previous studies. This may be because of the strong association between SCLC and a history of heavy cigarette smoking, which was also associated with ILA development [19].

Finally, we explored the impact of adding radiation therapy to chemotherapy for patients with LS-SCLC and ILAs. The OS was similar between the two therapies, but the 2- and 5-year survival rates were considerably higher for the patients treated with CRT. This suggests that although there is a selection bias and risk of RRP for the patients with SCLC and ILAs treated with CRT, radiation therapy is probably necessary to achieve long-term survival.

The limitations of this study should be considered. First, the patients with LS-SCLC treated with early CCRT had milder ILAs that covered a lower proportion of the area of any lung zone compared with those treated with chemotherapy alone. Second, this was a retrospective study conducted at a single center that consisted of a Japanese-only population, and the generalizability of our findings to non-Japanese populations is unknown.

## Conclusion

A substantial proportion of patients with LS-SCLC had concomitant ILAs, which were a predictive factor for RRP in patients with LS-SCLC treated with CCRT. Therefore, clinicians should inform patients with LS-SCLC and ILAs that CCRT has both the possibility of long-term survival and risk of RRP. The future identification of patients with LS-SCLC and ILAs treated with CRT who are at high risk for RRP is essential, and a multicenter analysis of big data is warranted. This would allow many patients with LS-SCLC and ILA to safely receive CRT and achieve long-term survival.

## Supplementary Information

**Additional file 1: Fig. S1.** Typical computed tomography images of interstitial lung abnormalities (ILAs). (a) ILAs accounting for 5% of the area of a lung zone; (b) ILAs accounting for 10% of the area of a lung zone.

**Additional file 2: Table S1.** Comparison of baseline characteristics of patients with LS-SCLC between the ILA and non-ILA groups. ^a^Clinical staging was done according to the 7th edition of the TNM classification of lung cancer [9]. LS-SCLC, limited-stage small-cell lung cancer; ILA, interstitial lung abnormality; ECOG-PS, Eastern Cooperative Oncology Group performance status; V20, percentage of normal lung receiving at least 20 Gy.

**Additional file 3: Fig. S2.** Relationship between the incidence of RRP and V20. (a) Analysis of the association between the incidence of RRP and the lung V20 planned before radiation therapy (with RRP, n = 13; without RRP, n = 59). (b) Analysis of the association between the existence of ILA and lung V20 planned before radiation therapy (with ILA, n = 11; without ILA, n = 61). (c) Analysis of the association between the existence of ILA and MLD planned before radiation therapy (with ILA, n = 11; without ILA, n = 61). RRP, radiation-related pneumonitis; V20, percentage of normal lung receiving at least 20 Gy; MLD, mean lung dose. ■ Patients with ILA. *Patient where radiation therapy was stopped at 19.5 Gy due to RRP. ✕ Patients who developed RRP

## Data Availability

The datasets used and/or analyzed in the current study are available through the corresponding author on reasonable request.

## Abbreviations

ILA: Interstitial lung abnormality
LS-SCLC: Limited-stage small-cell lung cancer
CCRT: Concurrent chemoradiotherapy
ILD: Interstitial lung disease
RP: Radiation pneumonitis
CT: Computed tomography
NSCLC: Non-small-cell lung cancer
SCLC: Small-cell lung cancer
TNM: Tumor, nodes, metastasis
CRT: Chemoradiation therapy
GTV: Gross tumor volume
CTV: Clinical target volume
RRP: Radiation-related pneumonitis
PFS: Progression-free survival
OS: Overall survival
HR: Hazard ratio
CI: Confidence interval
MLD: Mean lung dose

## References

[1] Warde P, Payne D (1992). Does thoracic irradiation improve survival and local control in limited-stage small-cell carcinoma of the lung? A meta-analysis. J Clin Oncol.

[2] Pignon JP, Arriagada R, Ihde DC, Johnson DH, Perry MC, Souhami RL (1992). A meta-analysis of thoracic radiotherapy for small-cell lung cancer. N Engl J Med.

[3] Takada M, Fukuoka M, Kawahara M, Sugiura T, Yokoyama A, Yokota S (2002). Phase III study of concurrent versus sequential thoracic radiotherapy in combination with cisplatin and etoposide for limited-stage small-cell lung cancer: results of the Japan Clinical Oncology Group Study 9104. J Clin Oncol.

[4] Ohe Y, Yamamoto S, Suzuki K, Hojo F, Kakinuma R, Matsumoto T (2001). Risk factors of treatment-related death in chemotherapy and thoracic radiotherapy for lung cancer. Eur J Cancer.

[5] Washko GR, Lynch DA, Matsuoka S, Ross JC, Umeoka S, Diaz A (2010). Identification of early interstitial lung disease in smokers from the COPD Gene study. Acad Radiol.

[6] Jin GY, Lynch D, Chawla A, Garg K, Tammemagi MC, Sahin H (2013). Interstitial lung abnormalities in a CT lung cancer screening population: prevalence and progression rate. Radiology.

[7] Hatabu H, Hunninghake GM, Richeldi L, Brown KK, Wells AU, Remy-Jardin M (2020). Interstitial lung abnormalities detected incidentally on CT: a position paper from the Fleischner society. Lancet Respir Med.

[8] Putman RK, Hatabu H, Araki T, Gudmundsson G, Gao W, Nishino M (2016). Association between interstitial lung abnormalities and all-cause mortality. JAMA.

[9] Kobayashi H, Naito T, Omae K, Omori S, Nakashima K, Wakuda K (2018). Impact of interstitial lung disease classification on the development of acute exacerbation of interstitial lung disease and prognosis in patients with stage iii non-small-cell lung cancer and interstitial lung disease treated with chemoradiotherapy. J Cancer.

[10] Goldstraw P, Crowley J, Chansky K, Giroux DJ, Groome PA, Rami-Porta R (2007). The IASLC lung cancer staging project: proposals for the revision of the TNM stage groupings in the forthcoming (seventh) edition of the TNM Classification of malignant tumours. J Thorac Oncol.

[11] 113rd Turrisi AT, Kim K, Blum R, Sause WT, Livingston RB, Komaki R, et al. Twice-daily compared with once-daily thoracic radiotherapy in limited small-cell lung cancer treated concurrently with cisplatin and etoposide. N Engl J Med. 1999;340:265–71. 10.1056/NEJM199901283400403 9920950

[12] Le Pechoux C, Dunant A, Senan S, Wolfson A, Quoix E, Faivre-Finn C (2009). Standard-dose versus higher-dose prophylactic cranial irradiation (PCI) in patients with limited-stage small-cell lung cancer in complete remission after chemotherapy and thoracic radiotherapy (PCI 99-01, EORTC 22003-08004, RTOG 0212, and IFCT 99-01): a randomised clinical trial. Lancet Oncol.

[13] Collard HR, Ryerson CJ, Corte TJ, Jenkins G, Kondoh Y, Lederer DJ (2016). Acute exacerbation of idiopathic pulmonary fibrosis. An international working group report. Am J Respir Crit Care Med..

[14] Common Terminology Criteria for Adverse Events (CTCAE). https://ctep.cancer.gov/protocolDevelopment/electronic_applications/ctc.htm#ctc_40

[15] The Japanese Lung Cancer Society Guideline. https://www.haigan.gr.jp/modules/guideline/index.php?content_id=3

[16] Araki T, Putman RK, Hatabu H, Gao W, Dupuis J, Latourelle JC (2016). Development and progression of interstitial lung abnormalities in the Framingham Heart Study. Am J Respir Crit Care Med.

[17] Li F, Zhou Z, Wu A, Cai Y, Wu H, Chen M (2018). Preexisting radiological interstitial lung abnormalities are a risk factor for severe radiation pneumonitis in patients with small-cell lung cancer after thoracic radiation therapy. Radiat Oncol.

[18] Higo H, Kubo T, Makimoto S, Makimoto G, Ihara H, Masaoka Y (2019). Chemoradiotherapy for locally advanced lung cancer patients with interstitial lung abnormalities. Jpn J Clin Oncol.

[19] Washko GR, Hunninghake GM, Fernandez IE, Nishino M, Okajima Y, Yamashiro T (2011). Lung volumes and emphysema in smokers with interstitial lung abnormalities. N Engl J Med.

[20] Hurst JR, Vestbo J, Anzueto A, Locantore N, Mullerova H, Tal-Singer R (2010). Susceptibility to exacerbation in chronic obstructive pulmonary disease. N Engl J Med.

[21] Putman RK, Gudmundsson G, Axelsson GT, Hida T, Honda O, Araki T (2019). Imaging patterns are associated with interstitial lung abnormality progression and mortality. Am J Respir Crit Care Med.

[22] Whittaker Brown SA, Padilla M, Mhango G, Powell C, Salvatore M, Henschke C (2019). Interstitial lung abnormalities and lung cancer risk in the National Lung Screening Trial. Chest.

[23] Hunninghake GM, Hatabu H, Okajima Y, Gao W, Dupuis J, Latourelle JC (2013). MUC5B promoter polymorphism and interstitial lung abnormalities. N Engl J Med.

