# Supplementary figures and images for: Chemoradiotherapy for limited-stage small-cell lung cancer and interstitial lung abnormalities
Source: Radiat Oncol. 2021 Mar 17;16:52. doi: 10.1186/s13014-021-01780-y (PMC7972232; doi:10.1186/s13014-021-01780-y)

## Slide 1
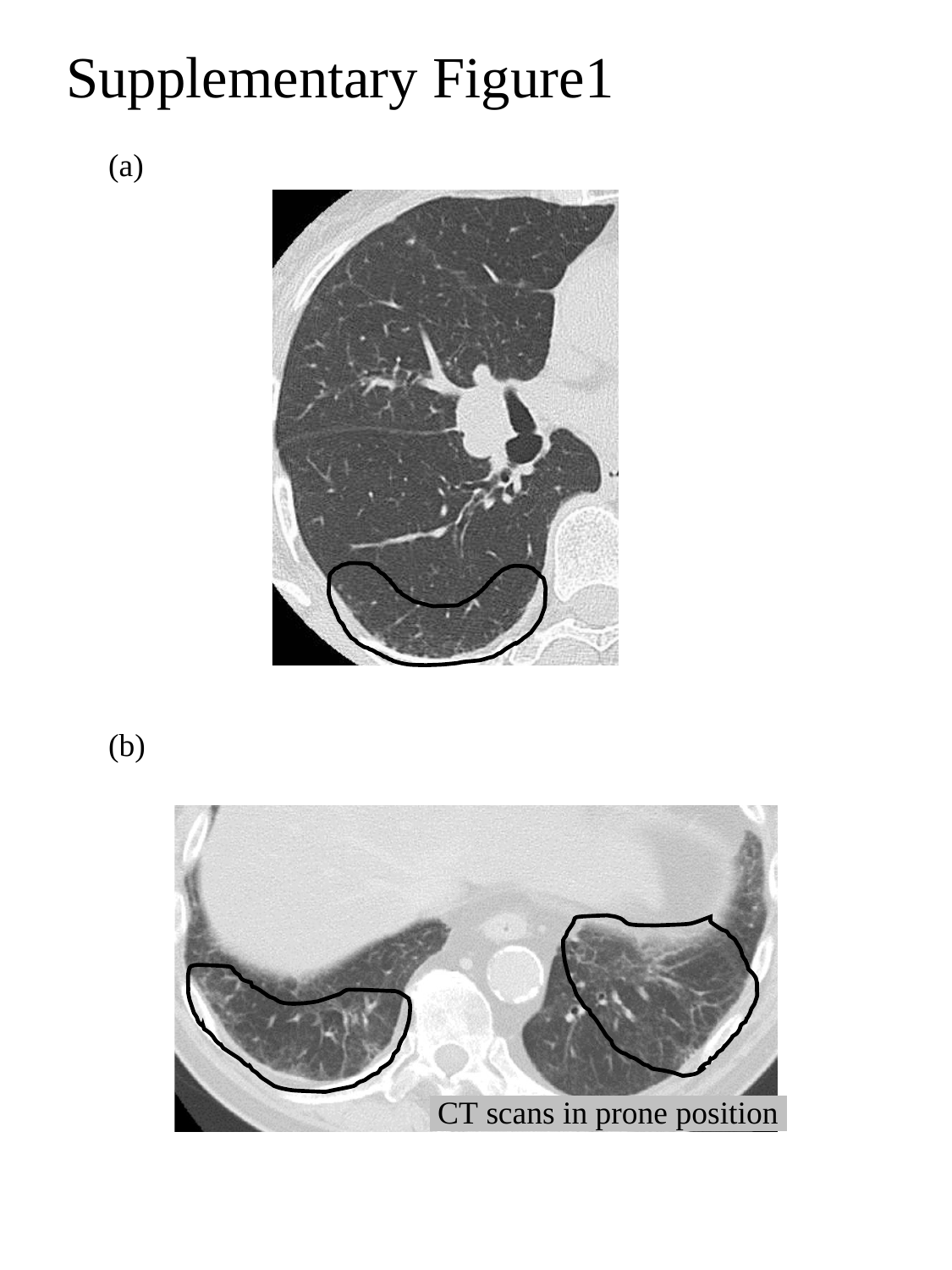

# Supplementary Figure1
(a)
(b)
 CT scans in prone position

Supplement: Supplementary file 1 — Additional file 1: Fig. S1. Typical computed tomography images of interstitial lung abnormalities (ILAs). (a) ILAs accounting for 5% of the area of a lung zone; (b) ILAs accounting for 10% of the area of a lung zone. [file 13014_2021_1780_MOESM1_ESM.pptx]

## Slide 1
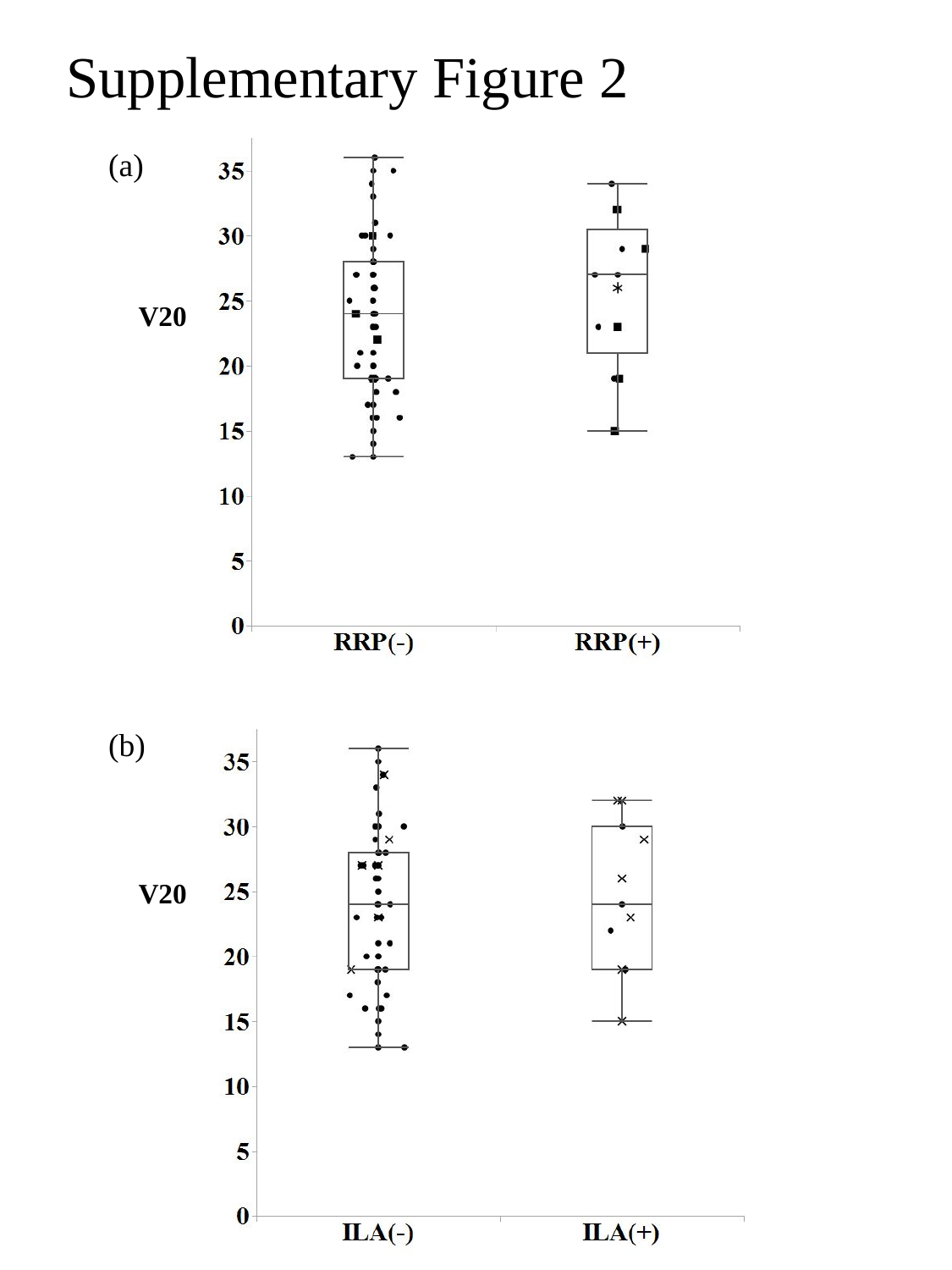

# Supplementary Figure 2
(a)
V20
(b)
V20

## Slide 2
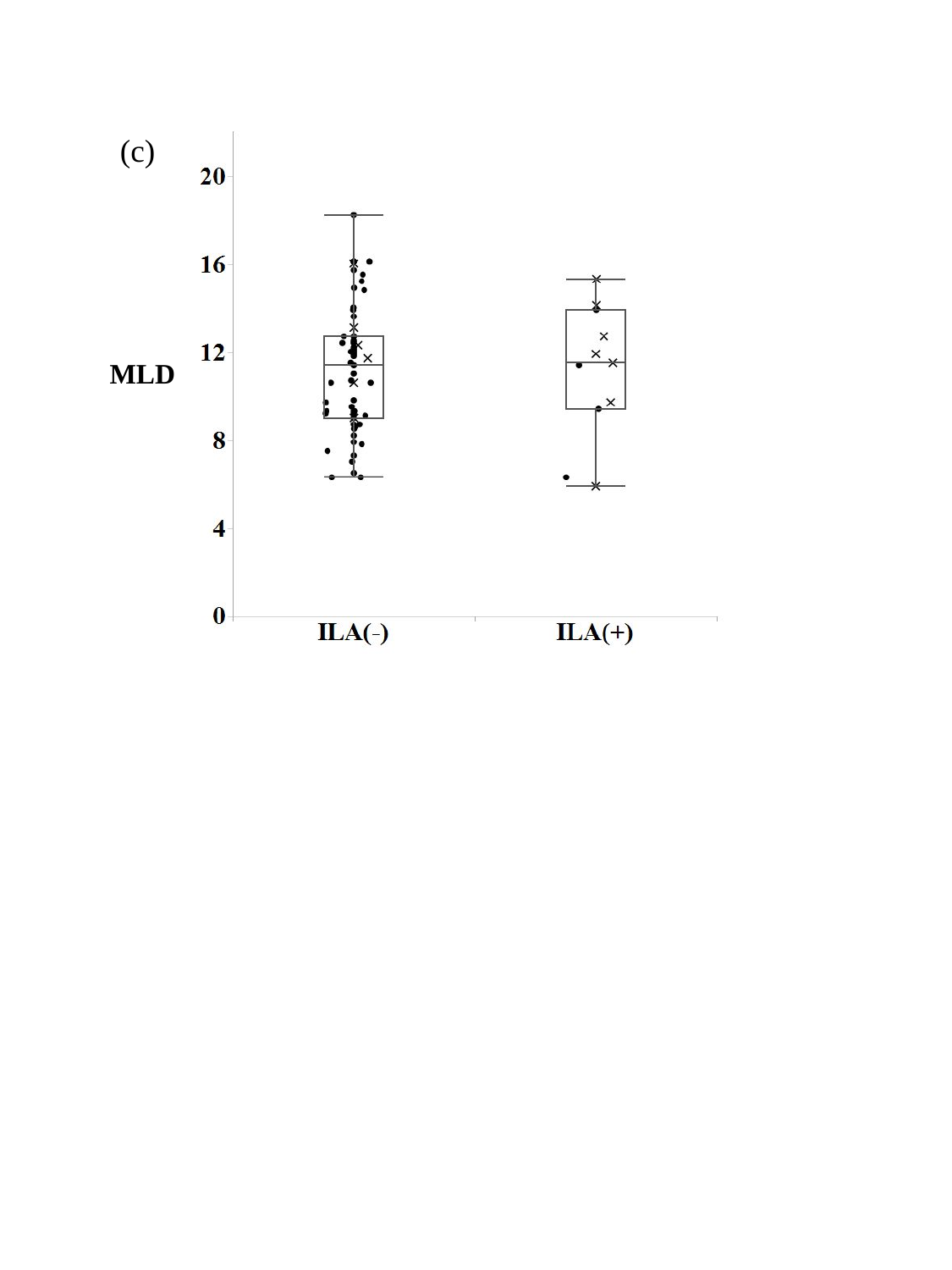

(c)
MLD

Supplement: Supplementary file 3 — Additional file 3: Fig. S2. Relationship between the incidence of RRP and V20. (a) Analysis of the association between the incidence of RRP and the lung V20 planned before radiation therapy (with RRP, n = 13; without RRP, n = 59). (b) Analysis of the association between the existence of ILA and lung V20 planned before radiation therapy (with ILA, n = 11; without ILA, n = 61). (c) Analysis of the association between the existence of ILA and MLD planned before radiation therapy (with ILA, n = 11; without ILA, n = 61). RRP, radiation-related pneumonitis; V20, percentage of normal lung receiving at least 20 Gy; MLD, mean lung dose. ■ Patients with ILA. *Patient where radiation therapy was stopped at 19.5 Gy due to RRP. ✕ Patients who developed RRP [file 13014_2021_1780_MOESM3_ESM.pptx]
