# Supplementary material for: Chemoradiotherapy for limited-stage small-cell lung cancer and interstitial lung abnormalities
Source: Radiat Oncol. 2021 Mar 17;16:52. doi: 10.1186/s13014-021-01780-y (PMC7972232; doi:10.1186/s13014-021-01780-y)
Supplement: Supplementary file 2 — Additional file 2: Table S1. Comparison of baseline characteristics of patients with LS-SCLC between the ILA and non-ILA groups. aClinical staging was done according to the 7th edition of the TNM classification of lung cancer [9]. LS-SCLC, limited-stage small-cell lung cancer; ILA, interstitial lung abnormality; ECOG-PS, Eastern Cooperative Oncology Group performance status; V20, percentage of normal lung receiving at least 20 Gy. [file 13014_2021_1780_MOESM2_ESM.docx]

**Supplementary Table 1. Comparison of baseline characteristics of patients with LS-SCLC between the ILA and non-ILA groups**

| Variable | ILA | Non-ILA | *P*-value |
| --- | --- | --- | --- |
|  | **(*n* = 11)** | **(*n = 62*)** |  |
| Age, ≥70/<70 years | 4/7 | 15/47 | 0.4611 |
| Sex, male/female | 10/1 | 45/17 | 0.2729 |
| ECOG-PS, 0/1/2 (0 vs. 1 or 2) | 7/4/0 | 35/24/3 | 1.0000 |
| Clinical stage^a^, I/II/III (II vs. III) | 0/0/11 | 0/15/47 | 0.1054 |
| Brinkmann index, ≥400/<400 | 11/0 | 60/2 | 1.0000 |
| V20, median (range) | 24 (15–32) | 24 (13–36) | 0.6435 |

^a^Clinical staging was done according to the 7th edition of the TNM classification of lung cancer [9]. LS-SCLC, limited-stage small-cell lung cancer; ILA, interstitial lung abnormality; ECOG-PS, Eastern Cooperative Oncology Group performance status; V20, percentage of normal lung receiving at least 20 Gy.
